# Emerging public health challenges during the COVID‐19 pandemic in Malawi: A review

**DOI:** 10.1002/puh2.40

**Published:** 2022-11-10

**Authors:** Isabel Kazanga Chiumia, Benjamin Azariah Mosiwa, Joe Nkhonjera, Betty Kazanga, Alistair Shingirai Mukondiwa, Aisha Twalibu, John Phuka, Don Eliseo Lucero‐Prisno

**Affiliations:** ^1^ School of Global and Public Health Kamuzu College of Health Science Lilongwe Malawi; ^2^ School of Social and Political Science University of Edinburgh Edinburgh Scotland; ^3^ Ministry of Health Phalombe District Hospital Phalombe Malawi; ^4^ IRD, INSERM Aix Marseille Univ, SESSTIM, ISSPAM Marseille France; ^5^ Schwarzman College Tsinghua University Beijing China; ^6^ Schulich School of Medicine and Dentistry University of Western Ontario Ontario Canada; ^7^ Department of Global Health and Development London School Hygiene and Tropical Medicine London UK

**Keywords:** COVID‐19, health policy, health systems, Malawi, pandemic, public health challenges, universal health coverage

## Abstract

The ongoing COVID‐19 pandemic has posed new and has aggravated already existing public health challenges in Malawi and worldwide. Having a better understanding of these challenges can help facilitate the identification of solutions and designing further public health interventions and policies for effective management of the COVID‐19 pandemic. This article presents an overview of the situation of COVID‐19 in Malawi and identifies emerging public health challenges that the country is facing amidst this pandemic. It is based on a review of relevant key policy documents, reports, and publications. Some of the key emerging challenges identified in Malawi are worsening population health and socio‐economic status; health system challenges like inadequate financing and human resources, disruption of essential health services; a rise in mental health conditions and suicide rates; teenage pregnancies and early marriages; and changes in some health policies. The findings point to the need to invest more in strategies for health promotion, health system strengthening and avoiding disruptions and recovery of services. These should include COVID‐19 vaccination promotion campaigns, improvement of the public health surveillance system, strengthening the health workforce, implementation of health financing strategies, procurement of adequate essential medicines and supplies, and strengthening of youth‐friendly reproductive health services, community health services and community engagement. These will ensure that the health system in Malawi is well‐equipped to deliver resilient, sustainable and quality health services amidst and beyond the COVID‐19 pandemic thereby promoting progress toward the achievement of Universal Health Coverage (UHC) and Sustainable Development Goals (SDGs) in Malawi.

## INTRODUCTION

The ongoing COVID‐19 pandemic has posed new and has aggravated already existing public health challenges in Malawi and worldwide. While the pandemic in itself has been a disaster to humanity, it has exposed and created far‐reaching weaknesses and challenges in many health systems globally, including in Malawi. The pressure the pandemic has exerted on health systems has uncovered and worsened gaps in human resources, health financing, medical equipment, and essential drugs making it more challenging to manage other health conditions [1]. The pandemic has also exposed long‐ignored gaps in social protection, structural inequalities, emergency preparedness, and resilience of populations in the face of a public health emergency, therefore, posing an even greater urgency in addressing issues of Universal Health Coverage (UHC) [2]. This domino effect has alerted many policymakers to start looking deeper into some of the public health challenges that follow the COVID‐19 pandemic to generate evidence that would inform health systems' recovery strategies amidst and beyond the pandemic.

The crisis has also reduced the abilities of the United Nations (UN) member states to achieve the UHC and the Sustainable Development Goals (SDGs) [1, 2, 3]. Given that all SDGs are interconnected, evidence shows that they have all been impacted [1]. Significant strides that were made on health‐related SDG 3 have been affected, and there is a potential impact on life expectancy due to excess mortality [4]. This can be attributed to the pandemic's disruptions of essential health services and insufficient capacity to manage increased demand for healthcare, among other things. Developing countries, in particular, have been hit hard by COVID‐19 [5], with 28 low‐income countries feared to most likely fail to attain SDGs 1–4, 6, and 7 by 2030 due to the pandemic [6].

This article provides an overview of the COVID‐19 situation in Malawi, how the government is managing it, and the emerging public health challenges that the country is facing during the pandemic. The findings can be beneficial for designing further public health interventions and policies for effective management of the COVID‐19 pandemic now and in the future and for strengthening the health system to effectively manage other health conditions amidst and beyond COVID‐19. Additionally, having a better understanding of emerging public health issues can be essential in promoting population health and working towards the achievement of UHC and SDGs.

### Context

Malawi is a country in Southern Africa with a population of 17,563,749 people and an annual population growth rate of 2.9% in 2018 [7]. Malawi's population is composed of a slightly higher percentage of females (51%) than males (49%), and the vast majority of the population (84%) resides in rural areas [7]. The unadjusted fertility rate in Malawi is high at 4.2 children per woman [4]. The literacy rate in Malawi for people aged 5 years and over is estimated at 68.6% [7]. Malawi's GDP per capita is low at US$ 603 in 2020 and approximately 51.5% of the population is living below the poverty line [8], that is, <$1.90 per day. The maternal mortality ratio in Malawi remains high at 439 deaths per 100,000 live births in 2017 [9]. The under‐five mortality rate in Malawi is 41.6 deaths per 1000 births, whilst the infant mortality rate is 30.9 deaths per 1000 live births [9]. Life expectancy at birth was estimated at 64.26 for both sexes in 2019 [9]. The HIV pandemic has significantly contributed to this low life expectancy. Malawi has one of the highest HIV prevalence in both the African region and globally, with 9.6% of Malawian adults living with HIV/AIDS, of which 91% are virally suppressed [10]. The high HIV prevalence in Malawi places exceptional demand on the available scarce health resources. Furthermore, there is also a huge burden attributed to Tuberculosis (TB), Malaria, lower respiratory infections, diarrheal and psychiatric diseases, and non‐communicable diseases (NCDs) [11].

### COVID‐19 situation in Malawi

A “State of Disaster” was declared in Malawi by the President on 20 March 2020 after the World Health Organisation (WHO) declared COVID‐19 a pandemic on 11 March 2020 [12]. Malawi registered its first confirmed coronavirus case on 2 April 2020 [13] and as of 23 August 2022, there have been 87,763 cases of confirmed COVID‐19 and 2676 deaths have been recorded in Malawi [13]. In April 2020, Malawi's Minister of Health announced plans to implement a 21‐day national lockdown from 18 April to 9 May 2020 [14]. The lockdown measures included bans on public gatherings; and the closure of all schools, national borders, airports etc. [12]. The announcement of the lockdown triggered country‐wide protests, which led to its suspension, stating that a lockdown without financial support from the government to small businesses and poor households would have a devastating economic impact on millions of people [14]. In the early stages of the pandemic, the Malawi Government responded by appointing a Special Cabinet Committee on COVID‐19 which started its operation on 7 March 2020 to provide oversight, coordination, and direction for national COVID‐19 initiatives [14]. The multidisciplinary team was responsible for introducing and monitoring the implementation of critical policy decisions. Additionally, a National COVID‐19 Preparedness and Response Plan was initiated in early 2020. It was aimed at ensuring the prevention of the COVID‐19 spread and based on three scenarios; when there are no cases, when a case is confirmed, and when people are affected by COVID‐19 as clusters or with community transmission [10].

**FIGURE 1:**
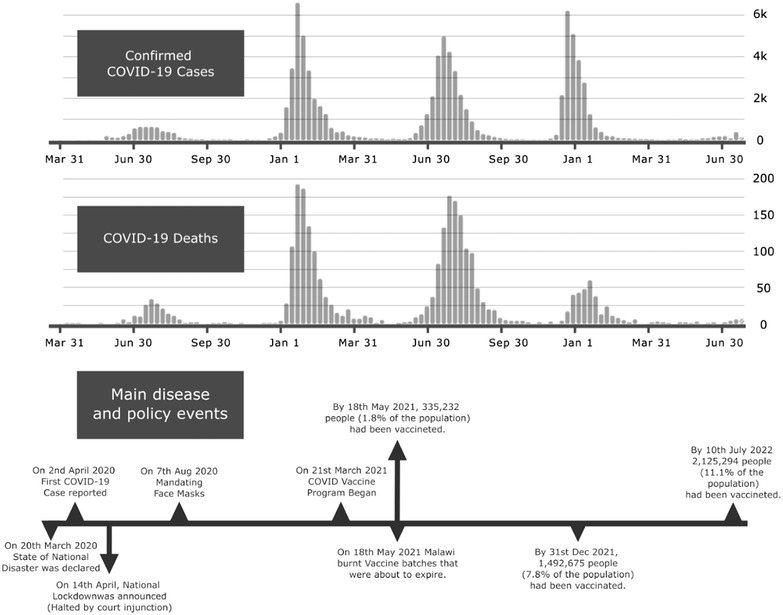
Covid 19 infections and deaths trends in Malawi (WHO, 2022)

Figure 1 presents the trends in COVID‐19 infections and deaths from March 2020 to June 2022 and highlights the major events at different times.

To slow down the spread of the COVID‐19 pandemic, Malawi, like other countries around the world, is enforcing various measures recommended by the WHO, which include but are not limited to the promotion of social distancing, regular handwashing with soap and water, use of hand sanitiser, practising respiratory hygiene, putting on masks, restriction of large gatherings, use of personal protective equipment (PPE), self‐isolation and quarantine of suspected or confirmed cases. The government also implemented policies on school closure, restrictions on international travel, mandatory COVID‐19 testing for international travel, reduced capacity on public transport, and working from home during the pandemic's peak. Furthermore, to prevent the spread of COVID‐19, the country is increasing its capacity to identify cases through surveillance, testing suspected cases, isolating and quarantining cases, and building capacity to manage severe cases that will require hospitalisation.

Malawi, like other countries, considers immunisation as a public health measure to prevent, contain and stop the transmission of the coronavirus. Through the Ministry of Health (MoH), Malawi rolled out COVID‐19 vaccines and they are currently being administered. Malawi rolled out its first COVID‐19 vaccine campaign in March 2021 using the AstraZeneca vaccine. A total of 360,000 doses of the vaccine were received from the COVAX facility which is a partnership between CEPI, Gavi, UNICEF and WHO received from [15]. In May 2021, Malawi was the first African country to burn expired COVID‐19 vaccines [16]. To increase vaccine uptake before the expiry dates, the government and its partners deployed fast‐track teams of community health workers to deliver COVID‐19 vaccines beyond health facilities nationwide to put vaccines closer to people who need them [16]. In August 2021, the Johnson & Johnson COVID‐19 vaccines donated by the U.S government through the COVAX facility were rolled out [16]. Malawi received 302,400 Johnson & Johnson vaccine doses making a total of 854,400 vaccine doses received through the UN‐let facility. Despite ongoing vaccine hesitancy as of 7 August 2022, Malawi had administered a total of 3,810,791 vaccine doses [17].

## METHODS

A review of literature on COVID‐19 in Malawi and globally was conducted to identify emerging public health challenges during the pandemic in Malawi. We examined peer‐reviewed articles, policy documents, reports, and guidelines. We searched PubMed, Medline, Lancet, and Google Scholar databases for articles published between December 2019 and February 2022. Search terms included “Covid‐19” OR “pandemic” AND “public health challenges” OR “universal health coverage” AND “COVID‐19” AND “health systems” OR “health policy” AND “preparedness and response” AND “Malawi.” Further articles were identified through a snowballing technique, where relevant cited literature from the papers was analysed was reviewed, with those matching the inclusion criteria selected for further analysis. We included only articles in English that discussed a combination of key concepts including public health challenges, COVID‐19, pandemic, health systems, policy, Malawi and health system strengthening, and literature on policy recommendations. We excluded articles that focused exclusively on previous outbreaks that were only primarily relevant before 2019 to ensure contextual relevance to the COVID‐19 pandemic. Finally, we supplemented our online literature search with a search of grey literature using similar search terms, including MoH reports and national strategic response plans, to analyse and give authentic reflections on the rapidly evolving landscape in the ongoing COVID‐19 pandemic.

## RESULTS

This review identified the following as some of the key emerging public health challenges during the COVID‐19 pandemic in Malawi:

### Population health and socioeconomic impacts

The COVID‐19 pandemic h had a devastating impact on the health and socio‐economic status of the population in Malawi. Research has shown that people at greater risk of being infected and dying from COVID‐19 are vulnerable groups such as individuals with pre‐existing conditions, the elderly, those living in poverty, women, children, immigrants, people living in crowded settlements, those in prison, and those who have been forcibly displaced [3]. Since the emergence of the pandemic, many people have gotten sick, suffered and died from COVID‐19‐associated illnesses.

The Malawi Confederation of Chambers of Commerce and Industry (MCCCI) reported that the country's economic growth would be affected because of disruptions to global trade and value chains, resulting in reduced demand for Malawi's export products, and tourism services [18]. They also estimated that 1.5 million jobs would be lost due to the pandemic. In addition, a study conducted by UNDP showed that 12% of the participants had lost jobs due to the pandemic [19]. Furthermore, the majority (77%) of the participants indicated that COVID‐19 had affected their income sources and levels, which was attributed to the closure of markets and businesses, and the loss of jobs.

Another study conducted in Malawi by the Governance and Local Development (GLD), reported that about 82% of the participants feared going hungry because of COVID‐19 and that most were going through financial hardships [20]. It has been recommended that the economic effects of COVID‐19 in Malawi necessitate cushioning measures such as those implemented in high‐income countries [21]. However, such measures are far‐fetched for a country like Malawi due to its financial constraints [22]. The Government of Malawi made efforts to cushion the economic shocks by extending its National Cash Transfer Programme to provide funds to cover 199,000 poor households in rural and urban areas at about MK 35,000.00 (approximately $45) per household, but the funds were only rated as adequate by 54% of the beneficiaries [23].

### Health financing challenges

Malawi's health system relies heavily on donor funding and Non‐Governmental Organisation (NGO) support [24] which is usually directed at specific health programs and related commodities [25]. COVID‐19 has significantly affected global and national economies through reduced productivity, business closures, and disruption of trade and tourism, thereby negatively impacting health financing [26]. Business closure has meant a reduction in the tax base and the failure of individuals to afford good healthcare (including private insurance). Dual health and global economic crises have affected the priority of international actors' aid to low‐ and middle‐income countries (LMICs). Although global aid for health increased from $40.4 billion in 2019 to $54.8 billion in 2020, the upward shift resulted from redirected resources towards the COVID‐19 response [27] while the other areas of health were underfunded.

Furthermore, the urgency in responding to COVID‐19 has caused a shift in financial distribution within national health priorities. Although the reallocation of resources during the pandemic may have been warranted, the consequences for some patient groups were dire [28]. For instance, disruption to the provision of maternal and child routine healthcare due to inadequate funding [29]. Another example of far‐reaching COVID‐19‐driven funding cuts is the reduction of Neglected Tropical Diseases and reproductive health funding by the United Kingdom government that impacted Malawi [30].

### Health workforce challenges

Barely a month after the first COVID‐19 case in Malawi, doctors and nurses began protesting citing poor working conditions, including the lack of PPE and low wages [31]. This impacted the availability and performance of health workers in all departments and at all levels, which compromised the fight against COVID‐19 and many other diseases.

Health workers also experienced different health effects and high workloads during the COVID‐19 pandemic. A study conducted in Blantyre city in Malawi revealed a 12.3% seroprevalence for SARS‐CoV‐2 among health workers from within the district [32]. Infected health workers meant fewer people were available to work in the ward, exerting more pressure on an already human resource‐deprived health system. This workload pressure, coupled with the virulence of COVID‐19, the lack of proper protective equipment and the community stigma attached to COVID‐19 were reported to have had significant mental health effects on front‐line healthcare workers [33]. In addition, health workers are susceptible to mental stress due to the pain of losing patients and colleagues to COVID‐19. The psychological well‐being of health workers is crucial for them to effectively discharge their duties. However, evidence shows that COVID‐19‐related anxiety interfered with health workers' performance and reduced their self‐efficacy. A study by Chorwe found that 25.5% of respondents had COVID‐19‐related anxiety and 48% functional impairment [34].

### Disruption of essential health services

There have been concerns that the provision of health services and surveillance has been negatively affected in Malawi and globally, either due to shifted priorities in health financing or other pandemic‐related reasons. A survey conducted by the WHO revealed that in about 90% of countries, there had been persistent substantial disruptions to essential health services [35]. In Malawi, it has been noted that the COVID‐19 pandemic affected HIV, TB, Maternal and Child Health, and NCD program services [36].

### HIV and TB services

The Malawi MoH set up real‐time monthly surveillance of HIV and TB activities from March 2020 to February 2021 in eight health facilities in Lilongwe, the capital city of Malawi, to assess the impact of COVID‐19 on HIV and TB services [37]. Despite MoH's guidance for HIV services during COVID‐19 stating that essential health services should continue [38], some HIV services were disrupted. The disruption partially led to a 39% overall reduction in HIV tests conducted [37], significant reductions in pre‐exposure prophylaxis (PrEP) initiation and use, and coverage of only 33% of the expected voluntary medical male circumcisions (VMMCs) [39]. According to Mbulaje et al. (2021), the disruptions in HIV services were mainly due to the reallocation of health workers, travel restrictions for community volunteers involved in HIV programs, discouraged hospital visits, interruptions to the supply of HIV commodities, and redirection of funds meant for HIV programs [36].

Research has also shown that COVID‐19 has affected the diagnosis and treatment of TB in Malawi. Since confirmation of COVID‐19 in Malawi, there has been an overall reduction in the number of persons presenting with presumptive TB (45.6%), bacteriologically diagnosed positive (2.6%), and those registered for TB treatment (19.1%) [37]. The reduction in TB notifications was attributed to COVID‐19 infection and COVID‐19 stigma because TB and COVID‐19 share similar symptoms, temporary facility closures, and inadequate PPE [40]. Despite the real‐time monthly surveillance, these declining trends in HIV and TB case detection were not addressed [37].

### Maternal and child health services

Malawi is among the eight countries in Sub‐Saharan Africa (SSA) experiencing significant and persistent disruptions to the continuity of maternal and child health services during the COVID‐19 pandemic [41]. This disruption is due to a decrease in mothers seeking care and restrictions imposed by hospitals on the number of patients allowed to attend antenatal care (ANC) at a time [42]. As a result, the number of institutional deliveries has reduced by more than 5% [41]. The quality of care given to mothers has been affected, mainly in terms of incomplete and late patient examination, leading to an increased likelihood of preventable and treatable complications going untreated [42]. The pandemic has also had a negative impact on routine vaccination of children younger than 1 year [43]. A study assessing childhood immunisation during the COVID‐19 pandemic in Haiti, Lesotho, and Malawi showed an early decline in vaccination uptake in all countries except Malawi, likely due to a lack of adherence to COVID‐19 restrictions in rural areas [43]. Furthermore, there was a more pronounced decline in vaccination in 2021, which corresponded with peaks in COVID‐19 infections that had higher cumulative cases and case fatality rates [43]. These significant declines in Malawi are likely due to increased restrictive measures and insufficient community outreach interventions during the waves; for instance, community outreach activities were suspended during the second and third waves, which caused fears of visiting health facilities [43].

### Other services

Other consequences of the COVID‐19 pandemic on the health system include but are not limited to the cancellation of elective surgeries and other non‐urgent procedures, suspension of planned insecticide‐treated nets distributions, interruption of the supply chain, redeployment of staff due to restrictions of travel, and the inability of routine health systems to generate updated information on service deployment and health investment [3].

### Mental health challenges

COVID‐19 has led to an increase in mental health issues like; anxiety, stress levels and worsening suicide rates in Malawi. Between April and September 2020, during a partial lockdown in Malawi, there was a rise in suicide cases, most of which were attributed to financial hardship due to a slowdown in economic activities [44]. Mental health challenges were also observed in students who feared future employment uncertainties and the effect of COVID‐19 upon completion of their studies [45].

### Increase in teenage pregnancies and early marriages

Teenage pregnancies and early marriages have been a long‐standing public health challenge in Malawi and the COVID‐19 pandemic has worsened the situation. During the pandemic, Malawi has experienced a rise in teenage pregnancies and early marriages attributed to the effects of the pandemic. For instance, a 5‐month long school closure of schools at the peak of the pandemic resulted in increased school dropouts [13]. A COVID‐19 rapid assessment on teenage pregnancies and early marriages led by the Malawi Government reported over 40,000 teenage pregnancies between March and July 2020, an 11% increase from the same period in 2019 [45, 46].

### Gender‐based violence (GBV) against women and girls

Emerging data and reports from organizations working on GBV indicate that all forms of violence against women and girls have increased during the COVID‐19 pandemic [8]. For instance, a third of women over the age of 15 have experienced domestic physical violence [47]. Furthermore, there has been a high prevalence of intimate partner violence, a consequence of lockdowns meaning that homes may not be the safest spaces for women and girls [48]. Intensifying economic and social stress, restricted movement, and social isolation have led to this increase in GBV cases [49]. While no causal studies on the impact of COVID‐19 on GBV have been conducted in Malawi, the available data on GBV cases from Malawi Police Service shows a 68% increase in reported cases of sexual and GBV between 2019 and 2020 in the periods January to May (from 3424 to 5067 reported cases) [19].

These forms of GBV have negative physical and mental health consequences that include physical injury, chronic pain, unwanted pregnancies, sexually transmitted diseases, anxiety, depression, post‐traumatic stress disorder, miscarriages, and poor child development [50]. The disruptions to health, other essential services, and GBV prevention and response programs have hampered access to these services by GBV survivors [51].

### Policy changes to the management of health services

In response to the COVID‐19 pandemic, the MoH made several policy changes in the form of implementation guidelines to ensure continued effective health service delivery in Malawi while following COVID–19 preventive precautions [38]. For example, the Ministry developed criteria for guiding the continuation or suspension of HIV services depending on whether a service is essential or otherwise. The affected services included; HIV testing, treatment, care and support, VMMC, condom distribution, PrEP, cervical cancer screening, TB preventive therapy, and patient support groups that involve gathering of people and active tracing via community visits [38, 52, 53]. These changes, though necessary in some cases, negatively impacted the provision of respective services.

## DISCUSSION

Our analysis has revealed multiple public health challenges that have emerged amidst the COVID‐19 pandemic in Malawi. The most pressing challenges identified in our review include worsening population health and socio‐economic status; health system challenges like inadequate financing and human resources, disruption of essential health services; a rise in mental health conditions and suicide rates; teenage pregnancies and early marriages; and changes in some health policies.

The results show that the impact of COVID‐19 on population health in Malawi has been significant with approximately 87,763 confirmed cases in 2 years and 4 months and 3% of these have died [14]. However, this could likely be an underestimation of the actual impact of the pandemic due to diagnostic and reporting challenges. Furthermore, results show that COVID‐19 aggravated financial hardship in many Malawian households [54]. The loss of jobs and closure of businesses means people's income streams have been heavily affected. This has been made worse by the rising prices of commodities due to the disruption in the global supply chain which has negatively affected Africa's economy by limiting its ability to participate in trade [52]. Unlike in high‐income countries, most middle‐ and low‐income countries lack access to financial resources to cushion the economic shock suffered by citizens, and the pandemic threatens the livelihoods and well‐being of many households [55]. The economic shock from COVID‐19 has also affected the way nations deal with the pandemic, with some African countries, including Malawi, relaxing preventive measures such as lockdowns because of their perceived impact on the economy and the general livelihood of the citizens [56, 57].

This study has revealed that the COVID‐19 pandemic has overstretched an already resource‐limited health system in Malawi that is highly dependent on donor aid. Due to insufficient financial resources, there has been a re‐channelling of funds for COVID‐19 interventions at the expense of reducing the delivery of other essential services and compromising the quality of the services [36]. This calls for the need for the government to implement effective health financing strategies, for example, allocating additional government funding to the health budget. This will help to strengthen the health delivery system and its recovery efforts.

The findings also show that COVID‐19 has affected the health workforce in Malawi with the current 1.48 health workers per 1000 population ratio representing a 48% deficit against its national targets [58]. With such a shortage of healthcare workers, the additional workload of managing COVID‐19 cases has posed a significant challenge due to splitting attention between competing interests. Furthermore, high rates of COVID‐19 seropositivity among health workers meant fewer people were available to work in the ward, exerting more pressure on an already human resource‐deprived health system. The Malawian health workforce has already registered high levels of dissatisfaction due to high workload, poor pay, a perceived lack of recognition, and limited career progression opportunities [59, 60], and increased workload has worsened morale. Policymakers should emphasise the allocation of financial resources to mental health services and staff support programmes targeting nurses during pandemics. There is a need to conduct future research on mental health interventions that might be used to assist nurses with COVID‐19‐related anxiety and functional impairment [34].

During the pandemic, Malawi's health system has suffered disruption of various health services such as HIV and TB services, maternal and child services etc. Results show that the number of people accessing HIV testing services, PrEP and VMMC has declined. These findings are consistent with those of Rick et al. [46], who reported that across the four continents of Asia, Latin America and the Caribbean, Europe and Africa, HIV testing and associated services significantly reduced despite different mitigating factors aimed at reducing the impact. Similar trends were also reported by UNAIDS in 16 out of 19 countries [61, 62]. In the case of Malawi, the decrease in HIV testing, PrEP access and VMMCs conducted as a result of a reduction in the number of people seeking health services due to travel caution and restrictions, and a shift in priorities that affected the allocation of health workers from non‐emergency services to COVID‐19 control. Despite the progress that Malawi has made over the years in scaling up HIV diagnosis and treatment, COVID‐19 is likely to continue disrupting the provision of HIV services in the coming years [63]. Consequently, due to disruptions in preventive and screening services, Malawi will most likely witness a rise in HIV incidence that may sabotage previous efforts and take a long time to rectify. The rise in new cases will put more pressure on the already limited resources amidst the pandemic, and we will in effect witness high rates of AIDS‐related morbidity and mortality.

Similar trends of disruption were witnessed in TB services in Malawi, where there have been significant drops in numbers of confirmed TB cases, bacteriological diagnoses and cases registered for treatment. The findings in Malawi are consistent with the trends in SSA, where TB treatment and prevention services have been widely affected by COVID‐19, leading to poor patient outcomes [64, 65]. The factors that have been attributed to the trends in SSA include lower drug supply and uptake, a decrease in the use of health services, declining screening and diagnosis, and increased substance use [66]. The situation in Malawi is of major concern because global TB programming has been negatively affected by COVID‐19‐fuelled disruptions. The STOP‐TB Partnership reports disruptions such as reduced rates of TB diagnosis, treatment initiation, and treatment completion, which may cause an excess of 6 million TB deaths by 2025 [67].

Malawi has made several commitments toward achieving UHC as outlined in SDG 3.8 [68]. Central to those commitments is the Essential Health Package (EHP), which was developed to optimise resources required to deliver essential health services to all effectively. The range of services covered by the EHP includes, but is not limited to, reproductive, maternal, neonatal, and child healthcare (RMNCH), nutrition, HIV and AIDS, malaria, TB, and vaccinations. With the widespread disruptions of healthcare delivery across the world due to COVID‐19, it may be necessary to repeat the Malawi Harmonized Health Facility Assessment (HHFA) which collected data to determine the availability, readiness, and quality of health service delivery in Malawi's health facilities in 2018–2019 [69]. This will be crucial to have a clearer picture of the impact of COVID‐19 on Malawi's progress in achieving UHC. However, currently, with the evidence already provided by the HHFA, government and private sector investment should be aimed at equipping healthcare providers with training, guidelines, equipment, essential medicines, and diagnostics required to deliver EHP services.

Furthermore, this review has revealed a significant rise in teenage pregnancies and early marriages during the pandemic in Malawi. According to the literature, complications of pregnancy and childbirth are a leading cause of mortality among young women aged 15–19. Currently, Malawi registers 439 maternal deaths per 100,000 live births, 15% of which are adolescents [70]. Similar trends in the rise in teenage pregnancies have also been reported in many parts of SSA [13]. These trends are a rising concern for most countries due to evidence demonstrating that adolescents are twice as likely to die during childbirth as women of 20 years and above [71]. Malawi already has one of the highest Maternal Mortality rates (MMRs) in the world, the rise of teenage pregnancies, therefore, makes it highly unlikely, if not impossible, to achieve the ambitious global target of 70 deaths per 100,000 births by 2030. Therefore, there is a need for the Malawi government to strengthen the delivery of youth‐friendly reproductive health services and community engagement to address issues of teenage pregnancies and child marriages.

According to the United Nations Population Fund, pandemics exacerbate domestic abuse and other forms of GBV [60]. During the Ebola outbreak for example, because of the economic effects of the outbreak, there was a high risk of sexual exploitation of women and girls [72]. Similarly, during the COVID‐19, Ebola and Zika outbreaks, women's access to sexual reproductive services and financial resources was affected [72]. The limited data or research on GBV during the COVID‐19 pandemic needs to be addressed if we are to understand the impact of COVID‐19 on GBV, the changing context and any gaps in capacity or services. The use of gender‐responsive budgeting to assessing the gender‐differentiated impact of recovery programmes, as is the case in Malawi, is a good practice that can be emulated in other countries facing similar challenges [73].

## CONCLUSION

This article has identified some of the emerging public health challenges during the COVID‐19 pandemic in Malawi that needs to be tracked and addressed. These include worsening population health and socio‐economic status; health system challenges like inadequate financing and human resources, disruption of essential health services; a rise in mental health conditions and suicide rates; teenage pregnancies and child marriages; and changes in some health policies. The Malawi government must address these challenges by investing more in strategies for health promotion, and recovery services. The strategies should include COVID‐19 vaccination promotion campaigns, strengthening the public health surveillance system for quality data, effective prevention and control of the pandemic. In addition, there is also a need to strengthen the health workforce through recruitment, training, protection and motivation of health workers; implementation of health financing strategies (for example allocating additional government funding to recovery efforts); procurement of adequate essential medicines and supplies, and strengthening of community health services, youth‐friendly reproductive health services and community engagement to address issues of teenage pregnancies, early marriages, GBV against women and girls and vaccine hesitancy. These will ensure that the health system in Malawi is well equipped to deliver resilient, sustainable and quality health services thereby promoting progress towards the achievement of UHC and SDGs in Malawi.

## AUTHOR CONTRIBUTIONS

Isabel Kazanga Chiumia and Benjamin Azariah Mosiwa conceptualized the idea of the paper. A data gathering and drafting group was composed of Joe Nkhonjera, Betty Kazanga, Alistair Shingirai Mukondiwa, and Aisha Twalibu. John Phuka and Don Eliseo Lucero‐Prisno III guided and supervised the team. All authors contributed to the analysis of the data and the revision iterations. Final rewriting and revision were done by Isabel Kazanga Chiumia, Benjamin Azariah Mosiwa, Betty Kazanga and Joe Nkhonjera. All co‐authors agreed to the final draft of the paper.

## CONFLICT OF INTEREST

Isabel Kazanga Chiumia and Don‐Eliseo Lucero‐Prisno III are Editorial Board members of Public Health Challenges and also co‐authors of this article. To minimize bias, they were excluded from all editorial decision‐making related to the acceptance of this article for publication.

## ETHICS STATEMENT

There is no need for ethical approval.

## Data Availability

No database or primary data was used in writing this paper.
